# Analysis of 24-Hour Blood Pressure Profile and Antihypertensive Therapy in Male Heart Transplant Patients

**DOI:** 10.3390/jcm14186590

**Published:** 2025-09-18

**Authors:** Wioletta Raczyńska, Alicja Radtke-Łysek, Michał Bohdan, Anna Frankiewicz, Wojciech Sobiczewski, Marcin Gruchała

**Affiliations:** First Department of Cardiology, Medical University of Gdańsk, 80-952 Gdańsk, Poland

**Keywords:** heart transplantation, hypertension, reverse dipping pattern of blood pressure

## Abstract

**Background**: Although there has been an improvement in the survival rates of patients following heart transplantation, many complications, such as hypertension, continue to develop. The aim of the study was to assess the 24-hour blood pressure profile and hypertension treatment among patients after heart transplantation and comparison to the control group. **Methods**: A retrospective data analysis included 26 male patients post-heart transplantation and 39 male patients in the control group. During a routine visit, the following data were collected: 24-hour blood pressure monitoring, laboratory tests, and medical history. **Results**: Hypertension was diagnosed in 76.9% of heart transplant recipients (HTXr) and in 56.4% of the control group (Cx). During the night-time rest period, diastolic blood pressure values ≥ 70 mmHg were observed in 76.9% of HTXr (vs. 33.33% Cx, *p* = 0.001). The average daytime systolic/diastolic blood pressure did not differ significantly between the groups. It was also observed that the groups differed in circadian blood pressure (Chi^2^ML = 15.87, *p* < 0.001), as there were significantly more reverse dippers in HTXr than in the control group (30.8% (8) vs. 10.3% (4)). The same proportions were also noted in HTXr and the control group in terms of isolated nocturnal hypertension. **Conclusions**: Heart transplant recipients require a tailored approach to hypertension management, including a variety of medications and appropriate chronotherapy.

## 1. Introduction

Heart transplantation is regarded as the gold standard for managing end-stage heart failure, capable of significantly prolonging life expectancy and enhancing the quality of life for patients with otherwise untreatable cardiac conditions. However, despite advances in modern pharmacotherapy and multidisciplinary care, patients who have undergone heart transplants continue to experience numerous complications, including hypertension. It is estimated that approximately 50–80% of individuals within this population exhibit elevated blood pressure levels, with some data indicating that up to 95% of patients are affected by arterial hypertension.

The development of hypertension following heart transplantation is a complex process involving a multitude of physiological, pharmacological, and environmental factors.

Immunosuppressive agents, especially calcineurin inhibitors such as cyclosporine and tacrolimus, as well as corticosteroids, stimulate the renin-angiotensin-aldosterone system, causing fluid retention and heightened sensitivity to catecholamines [1,2,3,4,5,6,7,8,9]. Post-transplant, the endothelium—the inner lining of blood vessels—ay exhibit dysfunction that results in diminished nitric oxide production and increased secretion of vasoconstrictive agents such as endothelins [10,11,12,13,14]. This imbalance fosters vasoconstriction and raises vascular resistance, thereby contributing to hypertension. Alternatively, elevated blood pressure and peripheral vasoconstriction may be attributable to denervation and subsequent reinnervation processes, which fundamentally alter cardiovascular homeostasis. Specifically, denervation of afferent pathways from low-pressure cardiopulmonary baroreceptors may elicit the initial rise in blood pressure observed in heart transplant recipients (HTXr), while subsequent cardiac sympathetic reinnervation—lacking parasympathetic counterbalance—may further promote hypertension development [15,16,17,18,19,20,21]. Abnormal profiles such as non-dipping or reverse dipping have been associated with adverse outcomes, including graft survival, cardiac allograft vasculopathy (CAV), and overall mortality. Therefore, assessing 24-hour blood pressure variability is crucial not only for adequate diagnosis and management of hypertension, but also for long-term prognosis in this high-risk population. It should be noted that the present study did not include longitudinal pre- and post-transplant blood pressure measurements. Our analysis was limited to post-transplant recipients compared with a control group.

Accurate diagnosis and effective treatment of hypertension are therefore essential responsibilities for the attending physician in the care of these patients.

## 2. Materials and Methods

### 2.1. Design and Participants

**Figure 1:**
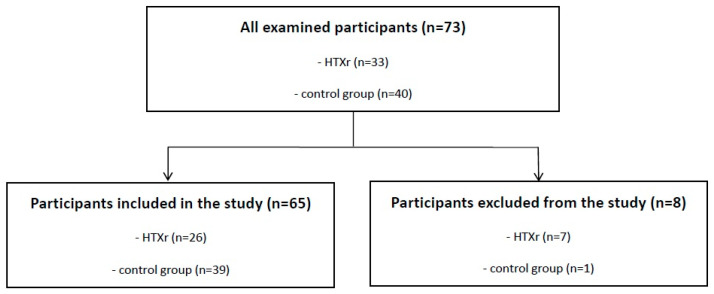
Study flow chart. HTXr, heart transplant recipient.

A retrospective analysis of data extracted from medical records encompassed 73 male participants, comprising 33 heart transplant recipients and 40 individuals serving as the control group without comorbid conditions. In the HTX group, the underlying etiologies of heart failure leading to transplantation were heterogeneous and included ischemic cardiomyopathy (*n* = 7), alcohol-related/environmental cardiomyopathy (*n* = 3), post-inflammatory cardiomyopathy (*n* = 3), familial/genetic cardiomyopathy (*n* = 9), and arrhythmogenic cardiomyopathy (*n* = 4). All patients in the HTX group had advanced heart failure and, prior to transplantation, were receiving full guideline-directed medical therapy, which typically included angiotensin-converting enzyme inhibitors (ACE inhibitors) or angiotensin receptor-neprilysin inhibitors (ARNI), beta-blockers, mineralocorticoid receptor antagonists, sodium-glucose cotransporter 2 inhibitors (SGLT2 inhibitors), and diuretics. Consequently, their blood pressure values before heart transplantation were typically low, reflecting both the severity of heart failure and the effects of pharmacological treatment. The control group consisted of male patients without a history of heart failure or heart transplantation, free of significant cardiovascular disease, and matched for age as closely as possible to the HTX group. Inclusion criteria stipulated an age range of 30 to 70 years and male sex. Exclusion criteria from the beginning included allograft dysfunction, valvular heart disease, other conditions requiring cardiac surgery, congestive heart failure classified as NYHA class III or IV, and chronic kidney disease at stages IV or V. In addition, during chart review, 7 patients from the heart transplant group and 1 patient from the control group were excluded due to unstable renal function with episodes of estimated glomerular filtration rate below 30 mL/min/1.73 m^2^. Finally, 26 HTXr and 39 controls were included in the analysis (Figure 1). Before performing ABPM, antihypertensive treatment was carefully optimized according to current clinical guidelines, in order to ensure reliable interpretation of blood pressure control and to avoid misclassification of resistant hypertension. The primary objective of the study was to evaluate the 24-hour blood pressure profile and hypertension management in post-heart transplant patients, with comparative analysis to the control group.

During routine visits, medical histories and laboratory samples were collected, and the general condition was assessed. Information regarding the date of heart transplantation, as well as current antihypertensive and immunosuppressive treatments, was obtained from individual medical records. The quantity and classification of antihypertensive and immunosuppressive medications were analyzed. Laboratory assessments encompassed blood count parameters, graft function indicators, electrolyte levels, lipid profile measurements, and blood glucose concentrations.

### 2.2. Blood Pressure Recordings

Ambulatory 24-hour blood pressure monitoring was conducted utilizing the SpaceLabs Model 90207 device (Spacelabs Inc., Richmond, Washington, DC, USA), employing the oscillometric technique. The data obtained included mean systolic and diastolic pressures over a 24-hour period, as well as mean pressures during daytime (6:00 AM to 10:00 PM, with measurements every 20 min) and nighttime (12:00 AM to 6:00 AM, with measurements every 30 min). This division of monitoring periods was adapted from O’Brien’s methodology. Additionally, the magnitude of the nocturnal dip was recorded. Hypertension diagnosed via ambulatory blood pressure monitoring (ABPM) was defined as daytime blood pressure exceeding 135/85 mmHg, nighttime blood pressure exceeding 120/75 mmHg, or 24-hour blood pressure exceeding 130/80 mmHg. Based on the percentage reduction in circadian blood pressure, patients were categorized as dippers (ΔSBP ≥ 10%), non-dippers (0 < ΔSBP < 10%), and reverse dippers (individuals exhibiting a nocturnal rise in SBP), in accordance with European Society of Hypertension (ESH) criteria [22,23].

### 2.3. Statistics

For comparisons of two qualitative variables, the following Chi-square tests, depending on table size, number of observations, and expected observations, were applied: the Maximum Likelihood Chi-square test (Chi^2^_ML_), Pearson’s Chi-square test, V-square test, and Yates’ corrected Chi- square test. The Shapiro–Wilk test was used to assess the normality of the distribution of quantitative variables, and Levene’s test was used to examine the homogeneity of variances. To determine whether quantitative variables differed between two groups, the following tests were applied: the independent samples Student’s *t*-test when the quantitative variable in both groups had a normal distribution and the variances were homogeneous, the Student’s *t*-test with variance estimation when the quantitative variable in both groups had a normal distribution but the variances were not homogeneous, and the Mann–Whitney U test when the quantitative variable in at least one group did not have a normal distribution. Effect size (Cohen’s d) was also calculated in variables that showed significant differences between groups, in order to present the strength of differences.

A repeated measures ANOVA was conducted to compare variables describing blood pressure simultaneously over time (during the day and the night) and between groups (HTXr and control group). Additionally, Tukey’s HSD test was employed for post hoc analysis.

Statistical analyses were performed utilizing TIBCO software (TIBCO Software Inc., Palo Alto, CA, USA ), as well as Statistica (data analysis software system, version 13). A *p*-value of less than 0.05 was regarded as statistically significant.

## 3. Results

A retrospective analysis of data collected from medical records encompassed 26 male recipients of heart transplants, with a mean age of 54.46 ± 10.51 years, and 39 male patients assigned to the control group (Cx), with a mean age of 57.61 ± 7.12 years. Both cohorts did not demonstrate significant differences in age, height, weight, or body mass index (BMI), as detailed in Table 1. Although recipients of heart transplants exhibited a higher prevalence of type 2 diabetes (HTXr 57.69% versus Cx 25.64%, *p* = 0.009), blood glucose and HbA1c (glycated hemoglobin) levels did not differ markedly between the groups, as shown in Table 2. The heart transplant group showed elevated levels of creatinine (HTXr 1.42 mg/dL versus Cx 1.09 mg/dL, *p* = 0.027) and triglycerides (HTXr 175.35 mg/dL versus 113.59 mg/dL, *p* = 0.001), alongside decreased levels of high-density lipoprotein (hdl) cholesterol (HTXr 45.92 mg/dL versus 58.98 mg/dL, *p* = 0.001) relative to the control group. No statistically significant differences were observed between the groups concerning C-reactive protein (CRP), fibrinogen, total cholesterol, and low-density lipoprotein (LDL) levels, as presented in Table 3.

The most frequently administered immunosuppressive medications among heart transplant recipients were tacrolimus (92.31%, *n* = 24) and mycophenolate mofetil (92.31%, *n* = 24). The average blood concentration of tacrolimus was 9.33 µg/L, with a standard deviation of ±3.65. Furthermore, 57.69% (*n* = 15) of the recipients were concurrently receiving prednisone.

Recipients of heart transplants and patients in the control group received comparable antihypertensive pharmacotherapy. The most frequently utilized antihypertensive agents within the studied populations included beta-blockers (HTXr 73.08% vs. Cx 64.10%, *p* = 0.452), calcium channel blockers (HTXr 42.13% vs. Cx 28.20%, *p* = 0.243), and ACE inhibitors (HTXr 57.69% vs. Cx 61.54%, *p* = 0.757). A striking difference was the high prevalence of loop diuretic use in HTXr (HTXr 61.54% vs. Cx 2.56%, *p* < 0.001), which may contribute to observed differences in BP control and metabolic profile (e.g., higher creatinine and triglyceride levels). Since antihypertensive treatment patterns may affect circadian blood pressure profiles, these results are presented here to provide context for the subsequent ABPM findings.

**Figure 2:**
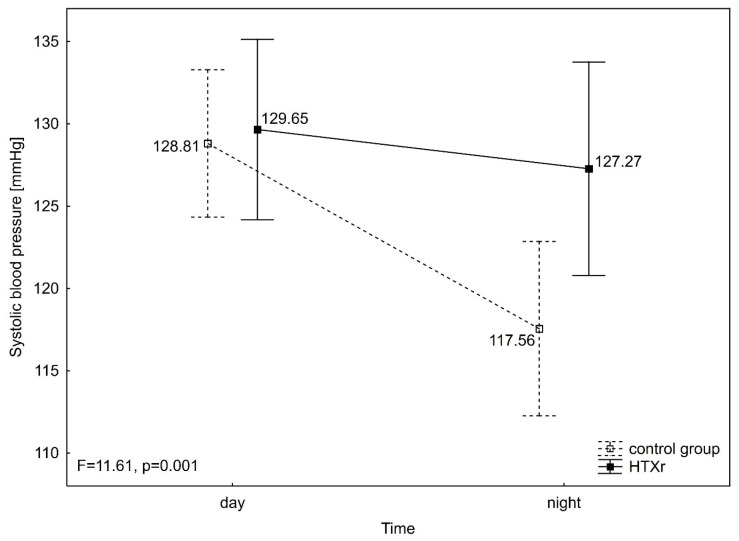
ANOVA with repeated measures for systolic blood pressure. HTXr, heart transplant recipient; SBP, systolic blood pressure.

Hypertension was diagnosed in 76.9% of the heart transplant patients (HTXr) and in 56.4% of the control group (Cx). An analysis of variance (ANOVA) with repeated measures revealed that systolic blood pressure (SBP) did not differ significantly between the groups (F = 2.09, *p* = 0.153) but varied significantly over time (F = 27.44, *p* < 0.001). Additionally, a significant interaction between group and time was observed (F = 11.61, *p* = 0.001). SBP was notably lower during the night compared to the day in the control group (SBP night 117.56 ± 15.79 mmHg vs. SBP day 128.81 ± 14.56 mmHg) (*p* < 0.001), whereas no significant difference was observed over time in the HTXr group (SBP night 127.27 ± 17.60 mmHg vs. SBP day 129.65 ± 13.04 mmHg). Furthermore, no significant differences were identified between groups when examined separately during the daytime and nighttime (Figure 2).

**Figure 3:**
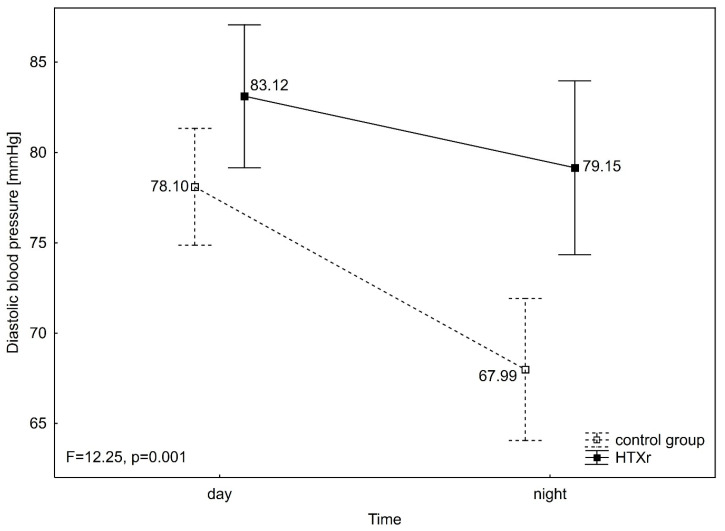
ANOVA with repeated measures for diastolic blood pressure. HTXr, heart transplant recipient.

**Figure 4:**
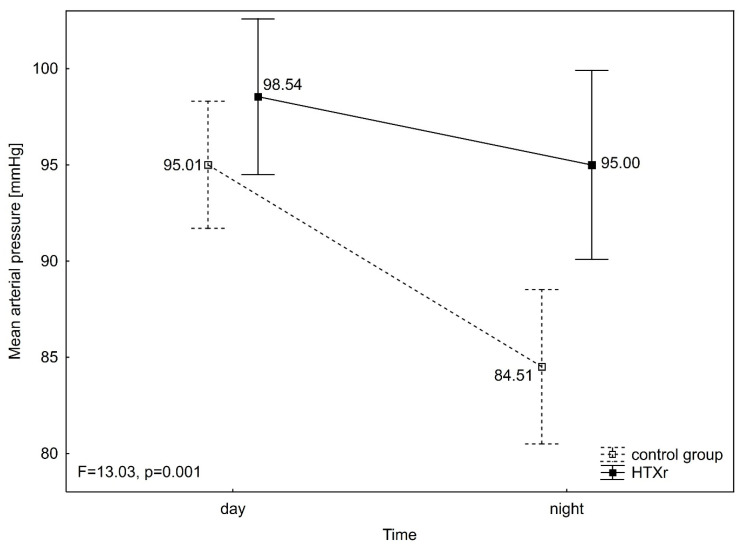
ANOVA with repeated measures for mean arterial pressure. HTXr, heart transplant recipient.

**Figure 5:**
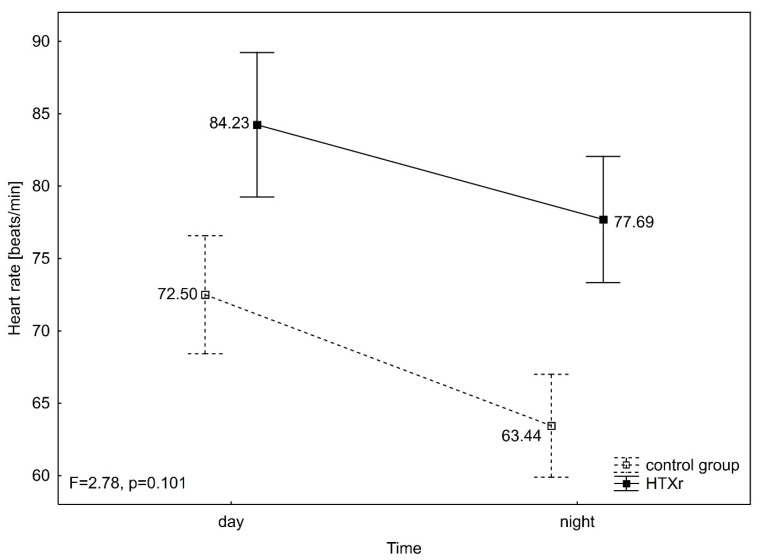
ANOVA with repeated measures for heart rate. HTXr, heart transplant recipient.

Diastolic blood pressure (DBP) differed significantly between groups (F = 8.93, *p* = 0.004), in time (F = 64.13, *p* < 0.001), and in interaction group over time (F = 12.25, *p* = 0.001). DBP was significantly lower during night than day in the control group (DBP night 67.99 ± 11.14 mmHg vs. DBP day 78.10 ± 10.33 mmHg) (*p* < 0.001), as well as in HTXr (DBP night 79.15 ± 13.85 mmHg vs. DBP day 83.12 ± 9.72 mmHg) (*p* = 0.025). Furthermore, significant differences between groups were observed in DBP during the night (*p* = 0.003), but not during the day (Figure 3). Mean arterial pressure (MAP) also differed significantly between groups (F = 6.53, *p* = 0.013), in time (F = 53.05, *p* < 0.001), and in interaction group over time (F = 13.03, *p* = 0.001). MAP was significantly lower during night than day in the control group (MAP night 84.51 ± 11.69 mmHg vs. MAP day 95.01 ± 10.64 mmHg) (*p* < 0.001), but not different in time in HTXr (MAP night 95.00 ± 13.73 mmHg vs. MAP day 98.54 ± 9.79 mmHg). Also, significant differences between groups were noticed in MAP during the night (*p* = 0.008), but not during the day (Figure 4). Heart rate (HR) differed significantly between groups (F = 19.66, *p* < 0.001), in time (F = 106.67, *p* < 0.001), but not in an interaction group x time (F = 2.78, *p* = 0.101). HR was significantly lower during night than day in both the control group (HR night 63.44 ± 11.43 mmHg vs. HR day 72.50 ± 14.13 mmHg) (*p* < 0.001) and HTXr (HR night 77.69 ± 10.61 mmHg vs. HR day 84.23 ± 10.26 mmHg) (*p* < 0.001). Also, HR was significantly higher in HTXr than in the control group during the night (*p* < 0.001) and during the day (*p* = 0.004) (Figure 5). It was also observed that the groups differed in circadian BP (Chi^2^_ML_ = 15.87, *p* < 0.001) as there were significantly more reverse dippers in HTXr than in the control group (30.8% (8) vs. 10.3% (4). The same proportions were also noticed in HTXr and control in terms of isolated nocturnal hypertension.

## 4. Discussion

### 4.1. Association with Drugs

Patients after heart transplantation often exhibit poorer BP control despite comparable pharmacotherapy. It has been identified that post-transplant patients predominantly utilize antihypertensive medications from the following categories: calcium channel blockers (CCB), angiotensin-converting enzyme inhibitors/angiotensin II receptor blockers (ACEi/ARB), and alpha-blockers. In selecting a specific therapeutic agent, both the drug’s impact on graft function and the potential for interactions with immunosuppressive medications must be carefully considered.

In our study, calcium channel blockers and angiotensin-converting enzyme inhibitors were the first-line agents in the treatment of arterial hypertension in patients after heart transplantation. Calcium channel blockers remain the most commonly used antihypertensive medications, partly due to the high frequency of renal dysfunction in the early post-heart transplant phase and concerns about potential hyperkalemia associated with the use of renin-angiotensin-aldosterone system (RAAS) blockers [24]. The benefits of using RAAS blockers are not fully understood in heart transplant patients. A recent study conducted by Masarone et al. [25] highlights the pleiotropic effects of ACE inhibitors in patients, including improvement of the peripheral vascular system, regulation of fluid and sodium balance, as well as the prevention of graft rejection and heart failure. The first randomized study comparing diltiazem and lisinopril as antihypertensive agents after heart transplantation revealed that both drugs were equivalently effective in achieving blood pressure control, and both were safe for treating hypertension after heart transplantation. Although titrated monotherapy with either of these drugs caused good blood pressure control in less than 50% of treated patients, it was suggested that a combination of both classes of drugs may result in larger effects [26].

Another group of medications frequently used in the studied HTXr group were beta-blockers. Similarly for non-transplant patients, beta-blockers are not regarded as first-line treatments for hypertension, although they may be beneficial when blood pressure is not well controlled with other therapies. A considerable number of patients required multi-drug regimens, and the common prescription of loop diuretics is indicative of the substantial burden of resistant hypertension among heart transplant recipients. Retrospective studies have demonstrated that beta-blockers are safe and particularly useful in heart transplant patients with resistant arterial hypertension [27]. Furthermore, the studied HTXr group showed statistically higher heart rate values than the Cx group. Abnormal heart rate control in patients after heart transplantation may be an independent factor affecting both graft and patient survival, and could contribute to the development of heart failure [28,29]. In our clinical practice, beta-blockers are avoided in the early years following heart transplantation and are only used in specific clinical scenarios, including atrial or ventricular arrhythmias and left ventricular dysfunction, particularly in the late phase post-transplantation [27].

In our study, we observed that loop diuretics were more frequently used in the HTXr patient cohort compared to the control group (HTXr: 61.54% versus 2.56%, *p* < 0.001). Diuretics are administered to manage fluid overload subsequent to cardiac transplantation. Cardiac transplant recipients exhibit abnormal responses to volume overload, demonstrating a propensity for salt and water retention despite a normal cardiac index.

Further therapies for blood pressure regulation may encompass alpha-adrenergic antagonists. These drugs can reduce blood pressure by diminishing peripheral vascular resistance and may result in a decrease in triglyceride (TG) levels, alongside an increase in high-density lipoprotein (HDL) cholesterol levels and the HDL cholesterol-to-total cholesterol ratio [30]. In our study, we observed significantly higher triglyceride levels in the group of heart transplant patients compared to the control group. According to the modified and revised Third Report of the National Cholesterol Education Program (NCEP-ATP III) [31] criteria, components of metabolic syndrome (MetS) include elevated TG levels, decreased HDL, presence of diabetes, elevated blood pressure values, or the use of antihypertensive medications. A retrospective study by Sponga et al. [32] demonstrated that the presence of MetS before and 1 year after heart transplantation (HTx) was associated with markedly worse survival, additionally identifying these factors as risk factors for mortality in univariate analysis. Furthermore, MetS 1 year post-Htx was linked to a significantly increased risk of developing cardiac allograft vasculopathy (CAV). The administration of alpha-blocker medications, which positively influence certain aspects of MetS, may potentially reduce the prevalence of metabolic syndrome among heart transplant recipients.

### 4.2. Association with ABPM Variability

Heart transplant recipients frequently present abnormal circadian BP patterns, including non-dipping and reverse-dipping profiles. From a therapeutic perspective, this highlights the importance of tailoring antihypertensive regimens to circadian variability. These abnormalities are associated with increased cardiovascular risk and graft dysfunction. However, data on interventions aimed at normalizing abnormal circadian blood pressure rhythms in heart transplant recipients are lacking. Clinically, evening dosing and prioritizing long-acting agents with sustained 24-hour efficacy (high smoothness index) may improve nighttime BP control more than conventional morning administration [33,34,35].

In our cohort, nocturnal hypertension and isolated nighttime BP elevation were markedly more frequent in HTXr than in controls, suggesting a high burden of masked hypertension. The high prevalence of masked hypertension in HTXr emphasizes the need for systematic use of 24-hour ABPM in follow-up, as office BP may underestimate risk.

### 4.3. New Therapy

In addition to the currently used treatment regimens, changing the timing of administering antihypertensive drugs should be considered. Introducing new antihypertensive medications in heart transplant patients to control blood pressure should be evaluated. One of the groups of drugs worth mentioning may be sodium-glucose cotransporter-2 inhibitors (SGLT2i). SGLT2 inhibitors have recently been recommended as an essential element of therapy for patients with heart failure and reduced ejection fraction (HFrEF) due to their favorable effects on mortality, clinical events, and quality of life [36]. Moreover, preliminary data indicate that the use of SGLT2 inhibitors appears to be well-tolerated and may provide benefits in both blood pressure and glucose level control in heart transplant recipients. A study by Kairo et al. [37] in patients with type 2 diabetes and uncontrolled nocturnal hypertension showed a significant reduction in both nighttime ambulatory systolic blood pressure and 24-hour ambulatory systolic blood pressure. Another retrospective study by Muir et al. [38] demonstrated that among 19 heart transplant patients treated with empagliflozin, systolic blood pressure was reduced by 12 mmHg (*p* = 0.03), and diastolic blood pressure by 7 mmHg (*p* = 0.03). In contrast, a study by Cehic et al. [39] involving 22 heart transplant patients with type 2 diabetes did not show a significant decrease in systolic blood pressure in patients treated with empagliflozin, although heart transplant patients treated with empagliflozin were more likely to use lower doses of diuretics after 12 months of treatment compared to patients that did not use empagliflozin. Both studies indicated that the use of SGLT2 inhibitors, particularly empagliflozin, appears to be safe and effective in treating selected heart transplant patients with diabetes.

We present the assessment of 24-hour blood pressure only in male heart transplant recipients. Currently, there are few studies evaluating blood pressure in heart transplant patients with gender stratification. Over the years, we have learned about numerous differences in macro- and microcirculation between men and women due to various physiological factors, including hormonal regulation. We believe that blood pressure assessment without gender stratification is inadequate, especially considering that women physiologically have lower blood pressure than men. None of our patients declared difference between gender identity and biological sex.

The most significant limitations of this study are its single-center, retrospective design. The results may not be as representative as those of multi-center reports; however, they could provide valuable data on a topic not extensively described in the literature—the assessment of 24-hour blood pressure profiles, variability (24-hour ABPM), and treatment in heart transplant patients, although frequently encountered in clinical practice. The true prevalence of hypertension in heart transplant patients is still unknown. Additionally, there is a continuing debate regarding optimal blood pressure goals and suggested pharmacologic regimens. Another important limitation is the absence of long-term outcome data, such as cardiac allograft vasculopathy (CAV) graft loss, or survival, directly linked to abnormal BP patterns. Prospective longitudinal studies are warranted to establish their prognostic relevance.

## 5. Conclusions

Arterial hypertension is very common after heart transplantation. Early diagnosis of arterial hypertension, conducting appropriate chronotropic therapy in hypertension considering the assessment of the hypotensive effect distribution of a given medication throughout the day (smoothness index), and implementing a therapy combining traditional antihypertensive treatment regimens using medications such as calcium blockers, ACE inhibitors/ARBs, and alpha-blockers in combination with new drugs such as SGLT2 inhibitors, may offer greater benefits in terms of better blood pressure control as well as lipid and glycemic management. Further analysis, possibly through a multi-center study, is needed to better explore the impact of this novel therapy. Clinicians should therefore prioritize early detection of abnormal circadian BP profiles using 24-hour ABPM and optimize therapy with long-acting antihypertensive agents to ensure effective 24-hour blood pressure control in heart transplant recipients.

## Figures and Tables

**Table 1:** Patient Demographic and Clinical Characteristics at Baseline.

Variables	HTXr (*n* = 26)	Cx (*n* = 39)	Test Results	*p*
	Mean ± SD			
Age [years]	54.46 ± 10.51	57.61 ± 7.12	−1.34 ^b^	0.189
Height [m]	1.75 ± 0.08	1.73 ± 0.05	1.19 ^b^	0.243
Body weight [kg]	81.50 ± 12.15	82.51 ± 9.87	−0.36 ^a^	0.719
BMI [kg/m^2^]	26.54 ± 3.42	27.55 ± 2.97	−1.24 ^a^	0.222
Tacrolimus level [ug/L]	9.33 ± 3.65	-	-	-
Time since transplantation [years]	4.90 ± 5.07	-	-	-

^a^ *t*-test; ^b^ *t*-test with variance estimation; BMI, body mass index; Cx, control group; HTXr, heart transplant recipient; SD, standard deviation.

**Table 2:** Patient Clinical Characteristics at Baseline.

Variables	HTXr (*n* = 26)	Control Group (*n* = 39)	Test Results	*p*
	% (*n*)			
Current smoking	8% (2)	5% (2)	0.00 ^c^	0.947
History of smoking	60% (15)	69% (27)	0.57 ^b^	0.452
History of cardiovascular diseases	42% (10)	82% (32)	10.73 ^b^	0.001
History of diabetes	58% (15)	26% (10)	6.77 ^a^	0.009
Percentage of patients taking statins	92% (24)	87% (34)	0.06 ^c^	0.806
Antihypertensive medications:			4.67 ^d^	0.197
does not take medications	4% (1)	18% (7)		
1 medication	19% (5)	21% (8)		
2 medications	54% (14)	33% (13)		
≥3 medications	23% (6)	28% (11)		
Beta-blocker	73% (19)	64% (25)	0.57 ^b^	0.452
α1-receptor blocker	8% (2)	0% (0)	1.05 ^c^	0.305
Calcium channel blocker	42% (11)	28% (11)	1.36 ^b^	0.243
ACE inhibitors	58% (15)	62% (24)	0.10 ^a^	0.757
Mineralocorticoid receptor antagonist (MRA)	0% (0)	5% (2)	0.19 ^d^	0.660
Sartans (ARBs)	19% (5)	5% (2)	1.93 ^d^	0.165
Loop diuretics	62% (16)	3% (1)	27.66 ^b^	<0.001
Thiazide diuretics	19% (5)	15% (6)	0.00 ^c^	0.946

^a^ Pearson’s Chi-square test; ^b^ V-square test; ^c^ Yates’ corrected Chi- square test; ^d^ Maximum Likelihood Chi-square test; HTXr, heart transplant recipient.

**Table 3:** Laboratory tests results.

Variables	HTXr (*n* = 26)	Control Group (*n* = 39)	Test Results	*p*
	Mean ± SD			
Creatinine [mg/dL]	1.42 ± 0.53	1.09 ± 0.17	2.22 ^b^	0.027
Glucose [mg/dL]	112.12 ± 22.85	111.41 ± 25.46	0.52 ^b^	0.601
TC [mg/dL]	184.19 ± 66.14	196.49 ± 49.26	−1.47 ^b^	0.141
LDL-C [mg/dL]	103.08 ± 53.99	115.17 ± 38.47	−1.77 ^b^	0.077
HDL-C [mg/dL]	45.92 ± 16.70	58.98 ± 16.12	−3.40 ^b^	0.001
TG [mg/dL]	175.35 ± 99.06	113.59 ± 59.02	3.21 ^b^	0.001
HbA1c [%]	6.05 ± 0.97	6.02 ± 0.93	−0.08 ^b^	0.938
Fibrinogen [G/L]	4.14 ± 1.40	3.73 ± 1.10	0.81 ^a^	0.421
CRP [mg/L]	5.04 ± 5.56	4.43 ± 7.25	1.27 ^b^	0.203

^a^ *t*-test; ^b^ Mann–Whitney U test; CRP, c-reactive protein; HbA1c, glycated hemoglobin; HDL-C, high-density lipoprotein; HTXr, heart transplant recipient; LDL-C, low-density lipoprotein; SD, standard deviation; TC, total cholesterol; TG, triglyceride.

## Data Availability

The original contributions presented in this study are included in the article. Further inquiries can be directed to the corresponding author.
